# Consensus-based guidelines for research in bracing in idiopathic scoliosis by the SRS comprehensive care committee in collaboration with SOSORT

**DOI:** 10.1007/s43390-026-01349-3

**Published:** 2026-07-01

**Authors:** Benjamin D. Roye, Alexander M. Park, Michael W. Brown, Mehdi M. Elfilali, Caglar Yilgor, Tom Schlösser, Sabrina Donzelli, Brian Smith, Stefano Negrini

**Affiliations:** 1 Columbia University Irving Medical Center, 622 W 168Th St, New York, NY 10032 USA; 2 Department of Orthopedics and Traumatology, Acibadem Mehmet Ali Aydinlar University, Istanbul, Turkey; 3 Department of Orthopedic Surgery, UMC Utrecht, Utrecht, Netherlands; 4 IRCCS Istituto Auxologico Italiano, Verbania, Italy; 5 Connecticut Children’s Medical Center, 282 Washington St, Hartford, CT 06106 USA; 6 Department of Biomedical, Surgical and Dental Sciences, University of Milan, Milan, Italy; 7 IRCCS Galeazzi S.Ambrogio Hostpital, Milan, Italy

**Keywords:** Bracing, Delphi, Idiopathic scoliosis, Research guidelines

## Abstract

**Purpose:**

Significant variability exists in the current literature of bracing research and outcome reporting, highlighting the need to standardize study design and reporting practices to ensure cross-study consistency and comparability. The purpose of this study is to develop updated consensus-based recommendations for designing, reporting, and publishing bracing research in pediatric patients with idiopathic scoliosis.

**Methods:**

An anonymous Delphi process and a final in-person meeting using the nominal group technique (NGT) established consensus-based guidelines among a multidisciplinary group of bracing experts. Three consecutive, anonymous Delphi rounds were administered to 30 multidisciplinary bracing experts invited to participate in this study. An 80% agreement threshold was set throughout the survey process and final in-person meeting. Items that did not reach consensus were refined to incorporate feedback and redistributed in subsequent rounds to improve consensus. Items that met consensus across all the rounds were simplified to remove redundant items to develop the final list of guidelines.

**Results:**

Of the 30 experts invited to participate, response rates were 24 (80%), 25 (83%), and 23 (77%) across survey rounds and 22 participants in the final, in-person meeting. Expert consensus was reached on 121 items across 6 domains which were refined and consolidated into a final 28-item summary of consensus-based guidelines for research in bracing idiopathic scoliosis (Fig. 1).

**Conclusion:**

Detailed, consensus-based research guidelines for bracing treatment in patients with idiopathic scoliosis were successfully developed. It must be acknowledged that these best practice guidelines are for research purposes only and are not clinical recommendations.

## Introduction

Bracing treatment is widely accepted as the primary non-operative treatment for idiopathic scoliosis (IS), [1–7] with the landmark BrAIST study demonstrating the clear effectiveness of brace treatment in decreasing risk of curve progression in adolescents with idiopathic scoliosis (AIS) and curves 20–40° and Risser 0–2 [8]. As a result, non-operative management has grown considerably, [9–11] with a nearly four-fold increase in bracing research over the past two decades.

Despite the clear utility of bracing in IS, the incredible growth of this literature is complicated by significant heterogeneity in the existing research [12–14]. A 2021 Scoliosis Research Society (SRS) survey of 218 members revealed considerable disagreement in clinical bracing practices, including radiographic timing, brace adequacy assessments, and criterion for brace discontinuation [14]. This variability is echoed in recent systematic reviews highlighting high risks of bias and inconsistent reporting of brace type, baseline curve magnitudes, and follow-up intervals [12, 13]. Therefore, there is an urgent need to standardize study design and reporting practices to ensure consistency and comparability across this growing body of literature.

In 2005, Richards et al. proposed the first guidelines for AIS bracing research through the SRS Committee on Bracing and Nonoperative Management [15]. Through a comprehensive literature review, their efforts sought to define consistent inclusion criteria and brace effectiveness. However, these guidelines were limited to AIS cohorts, lacked objective brace-wear metrics, and preceded the BrAIST study by almost a decade. Subsequently, Negrini et al. published updated guidelines through a collaborative effort between SOSORT and the SRS non-operative Committee in 2014 that broadened the target population to include all idiopathic patients and added recommendations on brace wear adherence data [16]. However, these recommendations are now over a decade old, and the evolving demands of current bracing practices and literature require updated guidelines. Technological advancements such as custom, three-dimensional braces, low-dose imaging, and widespread adoption of objective brace-wear monitors have reshaped bracing management, and emerging evidence has challenged historical curve magnitude recommendations for brace treatment [17–19]. Additionally, the growing recognition of the psychosocial and mental health impacts of brace treatment has improved patient care by recognizing that treatment adherence, body image, and well-being are intertwined [20].

As the innovative body of literature on bracing continues to expand, there is a growing need for more comprehensive and nuanced recommendations. We aim to update and expand upon the prior guidelines by offering more specific recommendations across key domains, including study methodology, brace wear prescriptions and compliance, study outcomes, patient-reported outcomes, skeletal maturity assessment, radiographic evaluation, and future directions. The objective of this study is to create consensus-based recommendations to serve as a framework for the design, implementation, and reporting of brace research in patients with idiopathic scoliosis. These updated recommendations will help ensure quality, facilitate cross-study comparability, and increase the potential for clinical applicability of future research findings in bracing studies.

## Methods

### Scoping literature review

A scoping review was conducted through PubMed to identify brace research recommendations in IS. Search terms included a combination of the keywords “idiopathic scoliosis,” “brace,” “bracing,” “research,” “guideline*,” “standard*,” and “recommendation*.” Titles and abstracts were screened to identify relevant articles, which were read in full. Results were limited to English studies involving IS in the pediatric population.

### Delphi process

The Delphi process was used to establish consensus among experts in the non-operative management of IS. The Delphi technique is a structured process that is used to reach expert consensus on complex issues through anonymous, multi-round questionnaires [21]. This process has been used by the authors in the past to establish best practices guidelines [22–26]. Participants responded to multiple rounds of anonymous questionnaires, and a core group of facilitators analyzed, summarized, and redistributed questions with controlled feedback. In each round, a 5-point Likert scale (“strongly disagree,” “disagree,” “neutral,” “agree,” “strongly agree”) was used to assess participant agreement on each item, and free-text responses were reviewed by the facilitators for integration into future iterative rounds.

### Participants

The SRS charged their Comprehensive Care Committee (SRS-CCC) with updating the brace research guidelines. An experienced facilitator (BDR) was appointed to lead this project, and five additional co-facilitators were selected from the SRS-CCC to guide this project based on their relevant clinical expertise, scholarly productivity, and leadership in the field. Members of the SRS-CCC nominated experts in non-operative management of scoliosis. After an initial screening to evaluate their qualifications as an expert, thirty orthopedic surgeons, clinicians, physiatrists, orthotists, physical therapists, and research scientists were invited to participate. Participation was completely voluntary and without pay. Participants (including all facilitators) were selected based on (4) specific inclusion criteria (Table 1).

**Table 1:** Expert inclusion criteria

Criterion	Description
1. Minimum Experience	At least 5 years of experience in orthotic management of idiopathic scoliosis
2. Clinical Practice Volume	Scoliosis represents a major component of clinical practice: (a) Approximately 4 or more patients per week (~ 200/year) for clinicians (b) Approximately 1 or more braces per week (~ 50/year) for orthotists
3. Membership	Membership of a major international society or study group, such as SOSORT, SRS, POSNA, EPOS, PSSG, and Harms
4. Scholarly Criterion	One or more of the following criteria within the past decade: (a) First or last author on a peer‑reviewed bracing publication (b) Podium presentation on bracing at a major international meeting (c) Grant funding from a recognized source for brace-related research

### Delphi rounds 1–3 (July 2025 – September 2025*)*

Initial survey items were created using the findings from the scoping literature review and revised by the co-facilitators before distribution. Items were categorized into the following themes: Study Methodology, Brace Wear Prescriptions and Compliance, Study Outcomes, Patient-Reported Outcomes, Skeletal Maturity, Radiographs, and Future Directions. Items that reached consensus (≥ 80%) and rejection (≤ 20%) were accepted into the final recommendations and removed from future rounds; items in equipoise (between 20 and 80% agreement) were modified to incorporate groups feedback before redistribution in subsequent rounds. Rounds 1–3 of the survey were distributed to participants in July, August, and September 2025, respectively. All questionnaires were created and distributed through Qualtrics (Qualtrics, Provo, UT).

### Final in-person meeting

A single facilitator (BDR) led and moderated the final in-person meeting at the 60th Annual SRS Meeting in September 2025 and presented the findings from the Delphi process using the nominal group technique (NGT). The NGT is an alternative face-to-face consensus method that promotes equal expert participation and is used to refine items in equipoise [27]. All experts were invited to contribute as equal participants, with a virtual option offered for those unable to join in-person [28]. Items near consensus were discussed in depth and voted on in real-time using an electronic audience response system, Mentimeter (Mentimeter, Stockholm, Sweden), with live results. This iterative process was repeated until items either reached consensus and were included in the final recommendations or were deemed that consensus would not be reached and excluded.

## Results

### Literature review

Through the scoping literature review, 47 articles were identified, however, only 2 were determined to be directly relevant to the scope of the study. These consisted of the previously proposed guidelines by Richards et al. and Negrini et al [15, 16].

### Participants

Of the 30 experts invited, 28 (93.3%) agreed to participate in this study. Overall, there were 14 surgeons, 2 physical medicine and rehabilitation physicians, 7 physical therapists, 4 orthotists, and 1 research scientist from the United States, Italy, Netherlands, France, United Kingdom, Canada, Greece, Hong Kong SAR, China, Australia, and Germany. Participants had a mean 37.5 ± 2.12 years in practice. Throughout the Delphi process, there were 24 (85.7%) respondents in Round 1, 25 (89.3%) respondents in Round 2, and 23 respondents in Round 3 (82.1%). Twenty-two participants, 13 in-person and 9 virtually, attended the final meeting.

### Consensus items

**Fig. 1:**
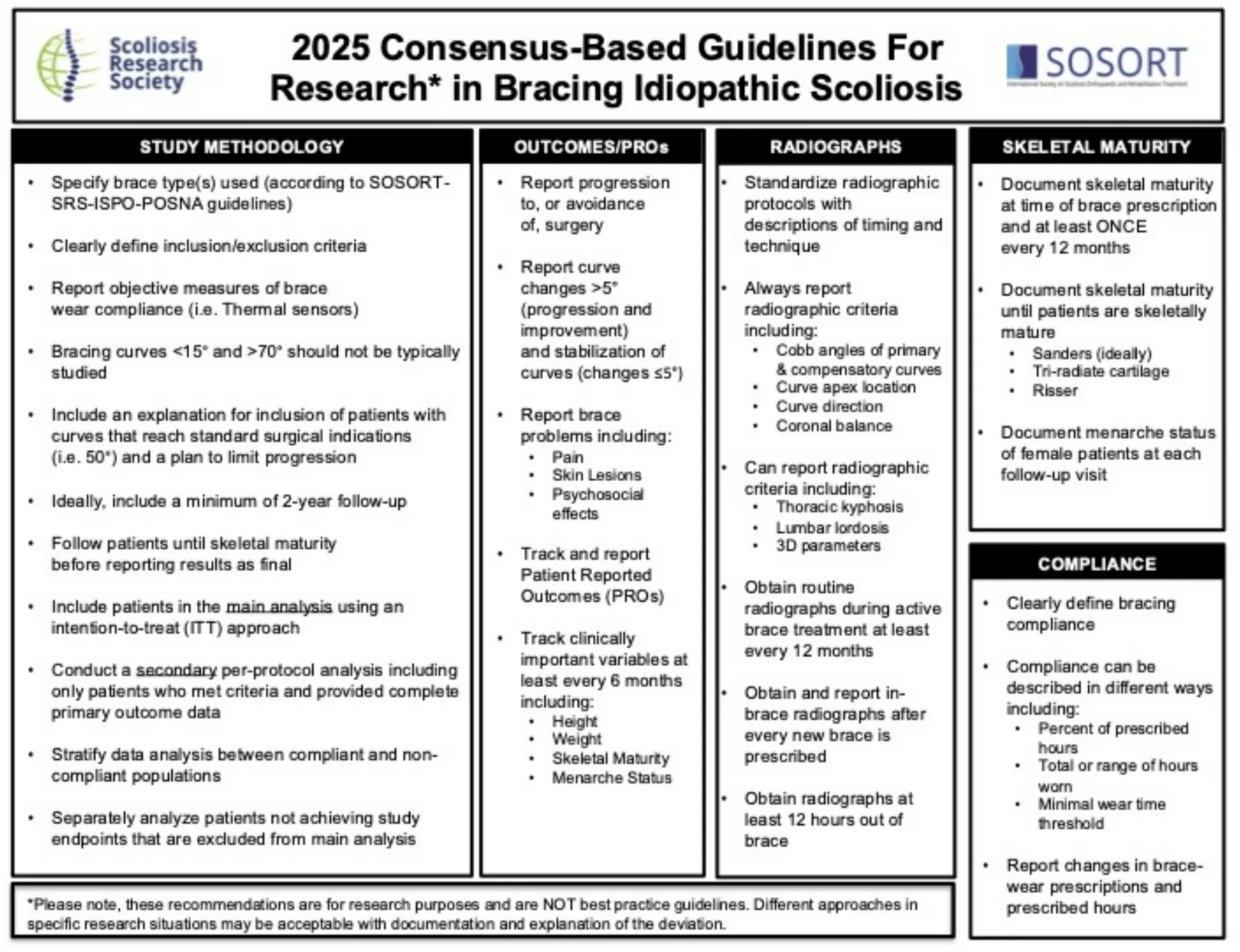
2025 Consensus-based guidelines for research in bracing idiopathic scoliosis

Throughout the Delphi process, the participants reached consensus on 121 items. During the final in-person meeting, an additional 8 out of the 12 proposed items achieved consensus. Redundant items were merged (i.e., “bracing curves < 10°…” was combined with “bracing curves < 15° should not typically be studied”), resulting in a final list of 66 items that achieved consensus (Table 2). The number of items proposed and those that achieved consensus across each round are presented in Table 3. The final 66 items that achieved consensus were reviewed by the co-facilitators and a focused framework with 28 items for research guidelines on bracing patients with IS is shown in Fig. 1. An additional 20 items reached consensus as potential future research topics throughout the study (Table 4).

**Table 2:** Final items that reached consensus

Study methodology
1. Research on idiopathic scoliosis should have a clearly stated question, hypothesis, and study endpoints
2. Research should have clearly defined inclusion/exclusion criteria including: (a) curve magnitude, (b) skeletal maturity, (c) brace-wear prescriptions, (d) age, (e) sex, (f) diagnosis, (g) curve location, (h) brace type, (i) comorbidities, and (j) previous treatment
3. There is a lower limit of curve size, below which studying bracing is not typically indicated
4. Bracing curves < 15 degrees should not typically be studied
5. Initiating bracing at Sanders 7/8 and Risser 5 are not typically indicated
6. Brace research must specify the specific brace type(s) used
7. Braces should be described by their classification according to SOSORT-SRS-POSNA
8. Brace patients should ideally be followed until skeletal maturity before reporting final results
9. Brace patients should ideally be followed for a pre-determined period of time after achieving skeletal maturity before reporting final results
10. Brace patients should ideally be followed for at least 2 years after skeletal maturity before reporting final results
11. A protocol standardizing follow-up time points for brace research should be developed
12. Growing patients should be evaluated clinically and radiographically at regular intervals
13. Routine follow-up radiographs should not be obtained more frequently than a specified time interval
14. Growing patients who are subjects in brace research should be evaluated clinically at a minimum of once every 6 months
15. Bracing curves > 70° should not be typically studied
16. Studies that evaluate bracing curves that reach standard surgical indications (i.e., 50°) need an explanation for including these patients and a plan to limit curve progression throughout the study
17. Objective measures of brace wear adherence reporting (such as thermal sensors) should be considered standard in scoliosis brace research
18. Patients not achieving study endpoints that are excluded from the main analysis should be analyzed separately
19. All patients should be included in the main analysis using an intention-to-treat (ITT) approach (i.e., analyzing all patients based on their initial group assignments, even those lost to follow-up)
20. A secondary per-protocol analysis may be conducted including only patients who met criteria and provided complete primary outcome data
Brace wear prescription and compliance
21. All research studies should clearly define bracing ‘non-compliance/non-adherence’ and ‘compliance/adherence.’
22. Non-compliance with brace treatment should be defined in brace research
23. Compliance/adherence can be defined as a % of prescribed hours (i.e., 80–100% = full compliance; 60–80% = partial compliance; and < 60% = non-compliance)
24. Brace prescriptions in brace studies should be categorized into standard ranges quantitatively (e.g., 18–23 h per day)
25. It is acceptable to report non-compliance/non-adherence based on a minimal threshold of wear time hours
26. All studies should stratify data analysis between ‘non-compliant/non-adherent’ and ‘compliant/adherent’ populations
27. In addition to analyzing these groups, it is important to report analyses using raw, quantitative data
28. Sub-analysis excluding non-compliant patients should be reported in brace research
29. The prescribed hours in brace should be reported in all brace research
30. Changes in brace-wear prescriptions should be tracked and reported
31. Differences in brace prescriptions should be controlled for in the analysis and study design
32. Brace compliance should be controlled for in the analysis and study design
Study outcomes and PROs
33. Clinically important variables to track at each visit should include: (a) height, (b) weight, (c) skeletal maturity, and (d) menarche status
34. Changes in Cobb angle are a critical outcome of brace treatment and must be reported
35. A change in Cobb angle should be ≥ 5 degrees to be considered a meaningful change
36. Definitions of treatment success and failure should be defined
37. Meaningful definitions of brace success include: (a) avoidance of surgery through the study period; (b) stabilization of the curve (change < 5°) through the study period; (c) improvement in curve magnitude (change ≤ 5°) through the study period
38. Meaningful definitions of brace failure include: (a) progression to surgery; (b) progression to meeting surgical indications; or (c) ≥ 50° Cobb angle
39. Negative outcomes of bracing other than curve progression are important to track and report, including non-compliance with brace prescription, negative psychosocial effects of bracing, pain related to brace wear, and skin lesions related to brace wear (bruising, skin breakdown, rashes, nodules, etc.)
40. Patient-Reported Outcome Measures (PROMs) should be tracked and reported in brace research
41. PROMs should be tracked at regular follow ups and trended throughout the study period
42. Research should report patient-reported outcomes using validated tools
43. It is preferable for patient-reported outcomes to be filled out by the patient rather than a caregiver surrogate
44. Brace modifications during the study period should be tracked and reported
45. Ideally, research on brace treatment should include a minimum of 2-year of follow-up
Skeletal maturity
46. It is important to measure skeletal maturity in brace research
47. Acceptable measures of skeletal maturity include: (a) Sanders, (b) Tri-radiate cartilage, and (c) Risser
48. Sanders is the ideal measure of skeletal maturity to be used for bracing research
49. Risser is an acceptable measure of skeletal maturity to be reported in bracing research in addition to other measures
50. Skeletal maturity needs to be documented at time of brace prescription
51. Ideally brace research should follow patients to skeletal maturity
52. Skeletal maturity needs to be documented until the measure is skeletally mature (e.g., Risser 5, Sanders 7–8)
53. Skeletal maturity should be captured at regular intervals, at least every 12 months
54. Growth should be tracked at each follow-up visit
55. Menarche status of girls should be tracked and reported
Radiograph**s**
56. Brace research projects should use, and describe, standardized radiographic protocols describing timing and technique of radiographs
57. Low dose scanning radiography is preferable to standard digital radiography, when available
58. The Cobb angle is the standard radiographic measure for evaluating brace effectiveness
59. Leg length discrepancies greater than a certain, pre-defined amount should be corrected with a lift for all radiographs (LLD > 2 cm)
60. The following radiographic criteria should *always* be reported in bracing studies: (a) Cobb of primary and compensatory curves, (b) curve apex location, (c) curve direction, and (d) coronal balance
61. The following radiographic criteria can be useful to report in bracing studies: (a) thoracic kyphosis, (b) lumbar lordosis, (c) 3D parameters, and (d) coronal balance
62. Out of brace radiographs should be obtained only after the patient has been out of brace for a minimum amount of time (≥ 12 h)
63. In-brace radiographs should be obtained, and reported, after every new brace is prescribed in brace research
64. It is acceptable to obtain in-brace radiographs after a certain amount of brace wear (e.g., not the day of delivery)
65. Bracing research should report the number of hours the patient is out-of-brace before their out-of-brace radiographs
66. Routine radiographs monitoring scoliosis during active brace treatment should be obtained at some regular interval, at least every 12 months

**Table 3:** Number of items that met consensus (≥ 80%) and rejection (≤ 20%) by round

	Delphi process						Nominal group process	
	Round 1		Round 2		Round 3		In-person meeting	
Items asked	170		115		51		12	
Consensus	Accepted	Rejected	Accepted	Rejected	Accepted	Rejected	Accepted	Rejected
Study methodology	22	3	8	3	8	0	0	0
Brace wear prescriptions and compliance	7	0	5	0	3	0	0	0
Study outcomes	20	0	3	0	2	0	0	0
Patient-reported outcomes	3	0	3	5	0	0	0	0
Skeletal maturity	6	0	2	0	1	0	3	0
Radiographs	15	4	5	0	0	0	5	0
Future directions	20	0	0	0	-	-	-	-
Total	93	7	26	8	14	0	8	0

**Table 4:** Topics for “Future Directions” of research that met consensus

FD1. Long-term outcomes such as prevention of adult-onset back pain related to scoliosis, and degenerative changes, are important goals of brace treatment
FD2. Identifying the best strategies for reducing or preventing the curve from getting worse, combining treatment and self-management approaches to avoid the need for surgery
FD3. Determining the effect of scoliosis and its treatment on quality of life
FD4. Determining the best ways to measure the effect of scoliosis and its treatment on quality of life in a way that is meaningful to patients
FD5. Identifying the best method of imaging scoliosis that also reduces exposure to radiation
FD6. Determining which type of brace is most effective in the treatment of early onset scoliosis
FD7. Determining which type of brace is most effective in the treatment of adolescent idiopathic scoliosis
FD8. Evaluating if earlier diagnosis of scoliosis changes choice of treatment and / or leads to better outcomes
FD9. Evaluating the psychological impacts (including on body image) of diagnosis and treatment of scoliosis
FD10. Determining the minimal amount of brace wear hours for a successful outcome
FD11. Developing clear guidelines for bracing
FD12. Developing reliable and effective tension monitoring
FD13. Determining if tension monitoring improves bracing compliance and / or outcomes
FD14. Identifying the best way to combine scoliosis specific physical therapy and bracing treatment to improve outcomes (including adherence, comfort, and overall correction)
FD15. Developing multicenter bracing registries
FD16. Evaluating the differences in patient outcomes when working with scoliosis specific orthotists for fabrication of braces as compared to non-scoliosis specific orthotists
FD17. Determining how or if brace patterns impact outcomes
FD18. Determining the value of brace wear during nighttime/supine positioning
FD19. Identifying the long-term psychosocial complications of full-time/part-time brace wear
FD20. Determining the 3D corrective effect of AIS bracing for specific brace types (including sagittal and axial)

Two main overarching recommendations were also agreed upon: 1) these guidelines refer only to research and not to clinical approaches, and 2) while it is reasonable to propose and conduct research projects that might deviate from these guidelines, any deviation should be explained and justified.

## Discussion

The goal of this study was to establish guidelines for designing, conducting, and reporting brace research in children with IS using a structured Delphi process and NGT. We established 66 final consensus items that were refined into a concise 27-item framework to serve as practical guidelines for future research efforts on bracing patients with IS (Fig. 1). Publishers may refer to these guidelines when determining the appropriateness of articles for inclusion in their respective journals, and future, prospective studies should aim to align with these consensus-based criteria to strengthen the quality and reproducibility of brace research. However, the proposed recommendations are designed to provide guidance for research purposes only and should not be interpreted as clinical best practice guidelines or immutable instructions. Therefore, while researchers must remain free in developing and testing their hypotheses, they are nevertheless requested to justify if, and when they do not follow these guidelines for any reason, whether voluntary (research hypothesis) or inevitable (e.g., retrospective data collection with unavailable information).

### Study methodology

When developing a bracing study for IS patients, studies should clearly define inclusion and exclusion criteria. Although previous guidelines have proposed specific inclusion criteria, these often limited the eligible patient population. Instead, we recommend that study cohorts be comprehensively characterized by age, sex, diagnosis, curve magnitude and location, skeletal maturity, previous treatment, brace type, and brace-wear prescription. The brace type should be specified in accordance with SOSORT-SRS-ISPO-POSNA guidelines [29]. Furthermore, we recommend that studies report objective measures of brace wear compliance, such as thermal sensors. While previous recommendations agreed on the importance of objective monitoring, they were limited in the technological and financial capabilities and availability of monitors at the time [15, 16]. However, since then, advancements in technology have addressed these previous barriers and now allow for the widespread use of robust compliance monitors for objective brace-wear assessment [19, 29, 30].

Curve magnitude is a central factor in brace treatment, and for bracing research purposes, we recommend excluding curves below 15° and above 70°. Although curves less than 25° in the past have fallen below the threshold for clinical intervention, there is a growing body of evidence that smaller curves, particularly in the JIS population where substantial growth potential remains, are excellent candidates for brace treatment [7, 31]. While the group felt it important to not exclude patients with curves that meet standard surgical indications of > 50°, there was consensus that they should not be greater than 70°. Additionally, the rationale for bracing any curve meeting surgical criteria (given the poor history of success in previous studies) should be explicitly justified, with inclusion of a plan to mitigate the risk of continued curve progression (i.e., more frequent clinical and radiographic evaluations), and the intention of prioritizing patient safety and well-being.

We recommend a minimum 2-year follow-up for studies reporting brace treatment outcomes, as this duration is standard for methodological rigor and comparability [32, 33]. However, shorter follow-up periods may be appropriate for specific endpoints, such as evaluating the effect of brace design on in-brace correction. Additionally, as skeletal maturity is a well-recognized factor in bracing research, and growth velocity varies considerably in this growing population, we recommend that patients are followed until skeletal maturity before reporting results as final [34].

Finally, in line with the original recommendations by Richards et al., the main analyses should follow an intention-to-treat (ITT) approach, reflecting real-world clinical practice and minimizes selection bias, as full patient compliance cannot be assumed [35]. However, because ITT analyses may dilute treatment effects by including noncompliant patients, secondary per-protocol (PP) analyses may be performed to assess outcomes among those who met inclusion criteria and completed the primary outcome measures. While PP analyses provide greater insight into treatment efficacy, they are limited by potential selection bias and reduced generalizability. The group reached consensus that patients who do not achieve study endpoints and are excluded from ITT analyses should be separately analyzed through secondary PP analyses, and that results should be further stratified by compliance status.

### Study outcomes and patient-reported outcomes

A main goal of non-operative management in IS is to delay or prevent curve progression to surgery. Therefore, studies should report both surgical progression and avoidance, along with curve improvement, stabilization, and progression, defined by a change in Cobb of ≥ 5°. This recommendation aligns with Richards et al.’s framework, where bracing success was defined as curve stabilization of ≤ 6°, and bracing failure was curve progression of ≥ 5°, along with the subsequent recommendation by Negrini et al. to also report curve improvement [15, 16, 36, 37]. While Cobb angle remains the standard outcome measure, other clinically important variables such as height, weight, skeletal maturity, and menarche status should be recorded at least every six months. Additionally, given the increasing importance of sagittal alignment in brace management, [38] thoracic kyphosis, lumbar lordosis, and three-dimensional parameters represent additional valuable radiographic parameters to monitor.

Furthermore, patient-reported outcomes (PROs) should be tracked and reported, as bracing affects one’s physical function, activity, self-image, and overall mental health [20, 39]. Therefore, it is equally important to report potential brace problems including pain, skin lesions, and psychosocial effects.

### Radiographs

Radiographic assessment in the non-operative management of children with IS should be carefully optimized to minimize unnecessary radiation exposure [40, 41]. To promote consistency and comparability, studies should standardize and clearly report radiographic timing, technique, and rationale. During active treatment, routine radiographs should be obtained at least every 12 months. Out-of-brace radiographs should be obtained at least 12 h after brace removal to avoid underestimating curve magnitude due to residual brace correction [42]. In-brace radiographs should be obtained and reported after each new brace prescription to assess spinal alignment and verify adequate correction [43].

Studies should always report core measurements including Cobb angles of primary and compensatory curves, curve apex location and direction, and coronal balance measurements. Thoracic kyphosis, lumbar lordosis, and three-dimensional parameters can additionally be useful to report comprehensive assessment.

### Skeletal maturity

Accurate assessment of skeletal maturity is pivotal in the non-operative management of IS as spinal curves progress most rapidly during periods of accelerated growth. In this growing population, we recommend that skeletal maturity be documented at the time of brace prescription and at least once every 12 months. The Sanders Skeletal Maturity Staging system has been shown to correlate more closely with growth velocity and curve progression than the traditional Risser sign [44–46]. Experts supported the use of the Sanders system, tri-radiate cartilage status, and Risser sign as acceptable methods for evaluating skeletal maturity. While the authors acknowledge that no current skeletal markers of maturity are perfect, during the final in-person meeting, experts identified Sanders as the currently ideal method. The Risser sign remains an acceptable measure of skeletal maturity but should be supplemented with additional measures to ensure comprehensive evaluation. These guidelines do not preclude utilizing additional means of assessing skeletal maturity.

### Compliance

Through this collaborative effort, consensus was achieved across a broad range of key areas relevant to non-operative IS management. However, challenges remained in establishing consensus on important definitions of “compliance” and “adherence” to brace prescription treatment. While the exact definitions of “compliance” and “adherence” were never established, there was strong agreement that researchers need to document brace exposure, including prescription, brace-wear prescription changes, and some element of wear time or compliance. Additionally, a variety of definitions of compliance were considered to be acceptable (Fig. 1), however, this list is not exhaustive. While the approach to defining and analyzing compliance may vary by investigator, these choices should be clearly described and justified within the study.

This study has several limitations. Although well established and extensively used in medical literature, the Delphi process and NGT are consensus methods that inherently depend on expert opinion, and their outcomes are influenced by participant composition. Experts were selected based on strict, well-defined criteria, and recommendations from the SRS Comprehensive Care Committee, which may have introduced potential selection bias.

The primary strength of this study is the establishment of a unified and structured framework to standardize future research projects and publications on bracing treatment of IS. These recommendations were developed through a rigorous, iterative Delphi process with stringent expert selection and were further refinement using NGT, incorporating perspectives from a multidisciplinary group of international experts. However, patient representatives were not included in the consensus process, and patient perspectives were therefore not directly represented.

Importantly, while many of the proposed elements align with sound clinical practice and may overlap with current best-practice guidelines, these recommendations are solely intended to guide research design and reporting [47]. Researchers may deviate from these recommendations for different reasons (research questions, available data, etc.), but they are requested to explain and justify their choices. Ultimately, these recommendations aim to strengthen methodological rigor, promote consistency across studies, and advance the broader goal of improving patient care through high quality evidence and more effective bracing practices.

## Data Availability

The de-identified, aggregate data supporting the findings of this study are available from the corresponding author upon reasonable request.

## References

[1] Karavidas N (2019) Bracing in the treatment of adolescent idiopathic scoliosis: evidence to date. Adolesc Health Med Ther 10:153–172. 10.2147/AHMT.S190565 31632169 PMC6790111

[2] Babaee T, Kamyab M, Ganjavian MS, Rouhani N, Jarvis J (2020) Success rate of brace treatment for juvenile-onset idiopathic scoliosis up to skeletal maturity. Int J Spine Surg 14(5):824–831. 10.14444/7117 33097584 PMC7671458

[3] Asher MA, Burton DC (2006) Adolescent idiopathic scoliosis: natural history and long term treatment effects. Scoliosis 1(1):2. 10.1186/1748-7161-1-2 16759428 PMC1475645

[4] van den Bogaart M, van Royen BJ, Haanstra TM, de Kleuver M, Faraj SSA (2019) Predictive factors for brace treatment outcome in adolescent idiopathic scoliosis: a best-evidence synthesis. Eur Spine J 28(3):511–525. 10.1007/s00586-018-05870-6 30607519

[5] Aulisa AG, Guzzanti V, Marzetti E, Giordano M, Falciglia F, Aulisa L (2014) Brace treatment in juvenile idiopathic scoliosis: a prospective study in accordance with the SRS criteria for bracing studies - SOSORT award 2013 winner. Scoliosis 9(1):3. 10.1186/1748-7161-9-3 24817906 PMC4016774

[6] Negrini S, Minozzi S, Bettany-Saltikov J et al (2015) Braces for idiopathic scoliosis in adolescents. Cochrane Database Syst Rev. 10.1002/14651858.CD006850.pub3 26086959 PMC10616811

[7] Bellamy M, Tung WS, Jayasuriya R et al (2025) Bracing effectiveness in idiopathic early onset scoliosis followed to skeletal maturity: a systematic review and meta-analysis. Spine Deform 13(3):939–950. 10.1007/s43390-025-01043-w 39841360 PMC12021973

[8] Weinstein SL, Dolan LA, Wright JG, Dobbs MB (2013) Effects of bracing in adolescents with idiopathic scoliosis. N Engl J Med 369(16):1512–1521. 10.1056/NEJMoa1307337 24047455 PMC3913566

[9] Tao L, Zhou S, Tao Z et al (2020) The publication trends and hot spots of scoliosis research from 2009 to 2018: a 10-year bibliometric analysis. Ann Transl Med 8(6):365. 10.21037/atm.2020.02.67 32355809 PMC7186647

[10] Luo C, Wu H, Liu W, Wong M (2024) A bibliometric review and visual analysis of orthotic treatment in adolescent idiopathic scoliosis from the Web of Science database and CiteSpace software. Medicine (Baltimore) 103(2):e36958. 10.1097/MD.0000000000036958 38215101 PMC10783366

[11] Xu J, Chen M, Wang X, Xu L, Luo X (2024) Global research hotspots and trends in non-surgical treatment of adolescent idiopathic scoliosis over the past three decades: a bibliometric and visualization study. Front Pediatr 11:1308889. 10.3389/fped.2023.1308889 38269292 PMC10806138

[12] Li X, Huo Z, Hu Z et al (2022) Which interventions may improve bracing compliance in adolescent idiopathic scoliosis? A systematic review and meta-analysis. PLoS ONE 17(7):e0271612. 10.1371/journal.pone.0271612 35857763 PMC9299303

[13] Costa L, Schlosser TPC, Jimale H, Homans JF, Kruyt MC, Castelein RM (2021) The effectiveness of different concepts of bracing in adolescent idiopathic scoliosis (ais): a systematic review and meta-analysis. J Clin Med 10(10):2145. 10.3390/jcm10102145 34063540 PMC8156678

[14] Halsey M, Dolan LA, Hostin RA et al (2021) Scoliosis Research Society survey: brace management in adolescent idiopathic scoliosis. Spine Deform 9(3):697–702. 10.1007/s43390-020-00265-4 33580371

[15] Richards BS, Bernstein RM, D’Amato CR, Thompson GH (2005) Standardization of criteria for adolescent idiopathic scoliosis brace studies: SRS Committee on Bracing and Nonoperative Management. Spine (Phila Pa 1976) 30(18):2068–2077. 10.1097/01.brs.0000178819.90239.d0 16166897

[16] Negrini S, Hresko TM, O’Brien JP, Price N, SOSORT Boards, SRS Non-Operative Committee (2015) Recommendations for research studies on treatment of idiopathic scoliosis: Consensus 2014 between SOSORT and SRS non-operative management committee. Scoliosis 10(1):8. 10.1186/s13013-014-0025-4 25780381 PMC4360938

[17] Cobetto N, Aubin CÉ, Parent S, Barchi S, Turgeon I, Labelle H (2017) 3D correction of AIS in braces designed using CAD/CAM and FEM: a randomized controlled trial. Scoliosis Spinal Disord 12(1):24. 10.1186/s13013-017-0128-9 28770254 PMC5525241

[18] Bagheri A, Liu XC, Tassone C, Thometz J, Tarima S (2018) Reliability of three-dimensional spinal modeling of patients with idiopathic scoliosis using EOS System. Spine Deform 6(3):207–212. 10.1016/j.jspd.2017.09.055 29735127

[19] Cordani C, Malisano L, Febbo F et al (2023) Influence of specific interventions on bracing compliance in adolescents with idiopathic scoliosis-a systematic review of papers including sensors’ monitoring. Sensors 23(17):7660. 10.3390/s23177660 37688117 PMC10490632

[20] Piantoni L, Tello CA, Remondino RG et al (2018) Quality of life and patient satisfaction in bracing treatment of adolescent idiopathic scoliosis. Scoliosis Spinal Disord 13(1):26. 10.1186/s13013-018-0172-0 30564635 PMC6295031

[21] Nasa P, Jain R, Juneja D (2021) Delphi methodology in healthcare research: How to decide its appropriateness. World J Methodol 11(4):116–129. 10.5662/wjm.v11.i4.116 34322364 PMC8299905

[22] Roye BD, Campbell ML, Matsumoto H et al (2020) Establishing consensus on the best practice guidelines for use of halo gravity traction for pediatric spinal deformity. J Pediatr Orthop 40(1):e42–e48. 10.1097/BPO.0000000000001379 30994582

[23] Negrini S, Grivas TB, Kotwicki T, Rigo M, Zaina F, international Society on Scoliosis Orthopaedic and Rehabilitation Treatment (SOSORT) (2009) Guidelines on “Standards of management of idiopathic scoliosis with corrective braces in everyday clinics and in clinical research”: SOSORT Consensus 2008. Scoliosis 4(1):2. 10.1186/1748-7161-4-2 19149877 PMC2651850

[24] Negrini S, Donzelli S, Aulisa AG et al (2018) 2016 SOSORT guidelines: orthopaedic and rehabilitation treatment of idiopathic scoliosis during growth. Scoliosis Spinal Disord 13(1):3. 10.1186/s13013-017-0145-8 29435499 PMC5795289

[25] Negrini S, Aulisa AG, Aulisa L et al (2012) 2011 SOSORT guidelines: Orthopaedic and Rehabilitation treatment of idiopathic scoliosis during growth. Scoliosis 7(1):3. 10.1186/1748-7161-7-3 22264320 PMC3292965

[26] Klineberg EO, Wick JB, Lafage R et al (2021) Development and validation of a multidomain surgical complication classification system for adult spinal deformity. Spine (Phila Pa 1976) 46(4):E267–E273. 10.1097/BRS.0000000000003766 33156283

[27] McMillan SS, King M, Tully MP (2016) How to use the nominal group and Delphi techniques. Int J Clin Pharm 38(3):655–662. 10.1007/s11096-016-0257-x 26846316 PMC4909789

[28] Smith D, Cartwright M, Dyson J, Aitken LM (2024) Use of nominal group technique methods in the virtual setting: a reflective account and recommendations for practice. Aust Crit Care 37(1):158–165. 10.1016/j.aucc.2023.09.004 37880060

[29] Negrini S, Aulisa AG, Cerny P et al (2022) The classification of scoliosis braces developed by SOSORT with SRS, ISPO, and POSNA and approved by ESPRM. Eur Spine J 31(4):980–989. 10.1007/s00586-022-07131-z 35190896

[30] Nakayama K, Kotani T, Kimura H et al (2021) The optimal anatomical position and threshold temperature of a temperature data logger for brace-wearing compliance in patients with scoliosis. Spine Surg Relat Res 6(2):133–138. 10.22603/ssrr.2021-0062 35478984 PMC8995123

[31] Whitaker AT, Hresko MT, Miller PE et al (2022) Bracing for juvenile idiopathic scoliosis: retrospective review from bracing to skeletal maturity. Spine Deform 10(6):1349–1358. 10.1007/s43390-022-00544-2 35852786 PMC9579105

[32] Montgomery F, Willner S, Appelgren G (1990) Long-term follow-up of patients with adolescent idiopathic scoliosis treated conservatively: an analysis of the clinical value of progression. J Pediatr Orthop 10(1):48–52 2298895

[33] Johnson TR, Segovia NA, Bryson X, Imrie MN, Vorhies JS (2024) Variations in duration of clinical follow-up after spinal fusion for adolescent idiopathic scoliosis: a survey of posna and srs membership. J Pediatr Soc North Am 5(3):645. 10.55275/JPOSNA-2023-645 40433342 PMC12088193

[34] Shi B, Mao S, Liu Z et al (2016) Spinal growth velocity versus height velocity in predicting curve progression in peri-pubertal girls with idiopathic scoliosis. BMC Musculoskelet Disord 17(1):368. 10.1186/s12891-016-1221-6 27562617 PMC5000496

[35] Molero-Calafell J, Burón A, Castells X, Porta M (2024) Intention to treat and per protocol analyses: differences and similarities. J Clin Epidemiol 173:111457. 10.1016/j.jclinepi.2024.111457 38977160

[36] Negrini S, Donzelli S, Negrini F, Arienti C, Zaina F, Peers K (2021) A pragmatic benchmarking study of an evidence-based personalised approach in 1938 adolescents with high-risk idiopathic scoliosis. J Clin Med 10(21):5020. 10.3390/jcm10215020. (**Published 2021 Oct 28**) 34768544 PMC8584294

[37] Aulisa AG, Toniolo RM, Falciglia F, Giordano M, Aulisa L (2021) Long-term results after brace treatment with progressive action short brace in adolescent idiopathic scoliosis. Eur J Phys Rehabil Med 57(3):406–413. 10.23736/S1973-9087.20.06129-8 32990686

[38] Matsumoto H, Warren S, Simhon ME et al (2020) It is not just about the frontal plane: sagittal parameters impact curve progression in AIS patients undergoing brace treatment. Spine Deform 8(5):921–929. 10.1007/s43390-020-00122-4 32338342

[39] Cheung KM, Cheng EY, Chan SC, Yeung KW, Luk KD (2007) Outcome assessment of bracing in adolescent idiopathic scoliosis by the use of the SRS-22 questionnaire. Int Orthop 31(4):507–511. 10.1007/s00264-006-0209-5 16896864 PMC2267629

[40] Knott P, Pappo E, Cameron M et al (2014) SOSORT 2012 consensus paper: reducing x-ray exposure in pediatric patients with scoliosis. Scoliosis 9(1):4. 10.1186/1748-7161-9-4 24782912 PMC4002921

[41] Luo TD, Stans AA, Schueler BA, Larson AN (2015) Cumulative radiation exposure with EOS imaging compared with standard spine radiographs. Spine Deform 3(2):144–150. 10.1016/j.jspd.2014.09.049 27927305

[42] Zaborowska-Sapeta K, Giżewski T, Binkiewicz-Glińska A, Kamelska-Sadowska AM, Kowalski IM (2019) The duration of the correction loss after removing Cheneau brace in patients with adolescent idiopathic scoliosis. Acta Orthop Traumatol Turc 53(1):61–67. 10.1016/j.aott.2018.10.001 30459102 PMC6424669

[43] Alvarez I, Poppino K, Karol L, McIntosh AL (2021) Lack of in-brace x-rays in compliant AIS patients wearing full-time TLSO braces associates with failure. J Orthop Surg Res 16(1):540. 10.1186/s13018-021-02650-9. (**Published 2021 Aug 31**) 34465348 PMC8406839

[44] Sanders JO, Khoury JG, Kishan S et al (2008) Predicting scoliosis progression from skeletal maturity: a simplified classification during adolescence. J Bone Joint Surg Am 90(3):540–553. 10.2106/JBJS.G.00004 18310704

[45] Risser JC (1958) The Iliac apophysis; an invaluable sign in the management of scoliosis. Clin Orthop 11:111–119 13561591

[46] Minkara A, Bainton N, Tanaka M et al (2020) High risk of mismatch between Sanders and Risser staging in adolescent idiopathic scoliosis: Are we guiding treatment using the wrong classification? J Pediatr Orthop 40(2):60–64. 10.1097/BPO.0000000000001135 31923164

[47] Roye BD, Simhon ME, Matsumoto H et al (2020) Establishing consensus on the best practice guidelines for the use of bracing in adolescent idiopathic scoliosis. Spine Deform 8(4):597–604. 10.1007/s43390-020-00060-1 32026441

